# Integrated multi-omics analysis indicates MARCO and ZBTB20 as candidate biomarkers in rheumatoid arthritis and tuberculosis

**DOI:** 10.3389/fimmu.2026.1886037

**Published:** 2026-08-31

**Authors:** Weihua Liu, Tiantian Ding, Keli He, Xu Han, Cantong Zhang, Yifang Mei

**Affiliations:** 1 Department of Rheumatology and Immunology, Shenzhen Third People’s Hospital, Shenzhen, China; 2 National Clinical Research Center for Infectious Diseases, Shenzhen Third People’s Hospital, Southern University of Science and Technology, Shenzhen, China

**Keywords:** immune infiltration, rheumatoid arthritis, single-cell RNA sequencing, biomarkers, tuberculosis

## Abstract

**Background:**

Rheumatoid arthritis (RA) patients have a clearly increased possibility to develop tuberculosis (TB). However, the common molecular background, useful diagnostic molecules, and treatment-related targets of this comorbidity are still not sufficiently explained. We attempted to search the shared genetic factors and candidate drug targets for RA and TB by an integrated multi-omics way.

**Methods:**

Bulk transcriptome data sets for RA and TB were obtained from Gene Expression Omnibus (GEO). Differential expression analysis and weighted gene co-expression network analysis (WGCNA) were first performed to collect overlapped disease-related genes. Least Absolute Shrinkage and Selection Operator (LASSO) regression was then applied to select hub genes. External data sets, single-cell RNA sequencing (scRNA-seq), and quantitative real-time PCR (qRT-PCR) using clinical samples were used for validation. We also carried out immune infiltration analysis, gene set enrichment analyses, molecular docking, and 100 ns molecular dynamics (MD) simulation, in order to describe possible function and treatment value of these genes.

**Results:**

A total of 312 co-expressed genes common to RA and TB were obtained, and these genes were mainly gathered in immune and inflammatory processes. *MARCO* and *ZBTB20* were kept as hub genes and showed acceptable diagnostic ability in RA (AUC = 0.839 and 0.795, respectively) and TB (AUC = 0.849 and 0.915, respectively). Single-cell results suggested their expression in T cells, B cells, and myeloid cell lineages, tightly correlated with immune infiltration and inflammatory signaling. Computational drug-repurposing analysis identified Tipifarnib as a candidate compound, and molecular docking and molecular dynamics simulations predicted potentially stable interactions with *MARCO* and *ZBTB20*. In the clinical cohort, *MARCO* expression was significantly higher in the RA-TB group than in healthy controls, whereas *ZBTB20* did not differ significantly among the four groups.

**Conclusions:**

Our study suggests that *MARCO* and *ZBTB20* may act as common diagnostic biomarkers for RA and TB. The clinical findings provide preliminary support for *MARCO* as a marker associated with RA–TB comorbidity, whereas the translational relevance of *ZBTB20* requires further validation. These findings provide a molecular framework for further investigation of RA–TB comorbidity and may inform the future development of tissue-specific biomarkers and targeted interventions.

## 1 Introduction

Rheumatoid arthritis (RA) is a systemic autoimmune disorder. Its main pathological features include long-term synovial inflammation, abnormal activation of the immune system, and slowly progressive destruction of joints (1). Genome-wide association studies (GWAS) have shown that RA has an obvious genetic component, and the heritability has been reported to be about 58% (2). In human genome, nearly 100 susceptibility loci related with RA have been described, and part of them are connected with disease severity (3). Although one risk allele usually has only a small influence, the combined effect of many genetic variations may greatly increase RA susceptibility (4). Therefore, RA development is not caused by a single reason, but by the joint action of genetic background, environmental exposure, sex-related factors, and some possible internal triggers. These factors together form a complicated pathogenic network, while many biological details still remain unclear.

In clinical treatment, tumor necrosis factor (TNF) pathway inhibitors are often used as immunomodulatory medicines for RA, but their use is associated with a higher risk of TB infection (5). TB is a major global health problem caused mainly by Mycobacterium tuberculosis (Mtb), and it is still one of the important causes of death worldwide. China is considered as a country with heavy TB burden, and epidemiological reports indicate around 800,000 new TB cases every year (6). From clinical view, 5-10% of people with latent tuberculosis infection (LTBI), in whom mycobacteria stay silently, may later progress to active tuberculosis (ATB) because of bacterial reactivation (7). In recent years, clinicians have reported more TB co-infection among RA patients (8). Previous studies suggested that RA patients may have 2–4 times higher TB infection risk than general population, and the standardized mortality ratio may reach 4.5 (9). Although the clinical connection between RA and TB has already been noticed, their shared genetic drivers and immune-metabolic pathways are still not described very well. Some earlier reports proposed several potential biomarker genes, such as IP10, Neopterin, CD64, and leucine-rich alpha-2 glycoprotein (10). Still, there are not enough studies about targeted treatment strategies for patients who have both diseases (11). For this reason, we used multi-omics data to explore these mechanisms.

In this study, common genes of RA and TB were investigated, with the purpose to understand the possible biological processes in RA complicated with TB. We further selected diagnostic markers from these common genes and evaluated their relationship with immune infiltration. Their possible value as biomarkers and therapeutic targets was also assessed.

## 2 Materials and methods

### 2.1 Data selection

Four microarray data sets were collected from GEO under the National Center for Biotechnology Information (NCBI) after systematic screening. The training group contained GSE77298 (GPL570) and GSE83456 (GPL10558). These data sets included transcriptomic profiles from 23 samples (7 healthy controls and 16 RA patients) and 153 samples (61 healthy controls and 92 TB patients), respectively. The validation group included GSE55235 (GPL96) and GSE19444 (GPL6947), containing 20 samples (10 healthy controls and 10 RA patients) and 33 samples (12 healthy controls and 21 TB patients). All series matrix files were processed by standard format. In addition, the RA single-cell data set GSE134420, with 4 samples having complete expression information, was downloaded. The TB single-cell data set PRJNA605083, including 7 samples with complete profiles, was also used for later analysis.

### 2.2 Differential expression analysis

The Limma package in R was used to perform microarray differential analysis. We compared disease and control samples in RA and TB data sets. By linear modelling and empirical Bayes moderation, this method can find genes with statistically meaningful expression changes. The threshold was set as |logFC| > 0.585, equal to 1.5-fold change, and adjusted p value < 0.05.

### 2.3 Weighted gene co-expression network analysis

Weighted gene co-expression network analysis (WGCNA) was conducted by the WGCNA-R package. This analysis was used to search gene modules with similar expression, to examine gene-disease associations, and to find possible central genes (12). The expression relationship was then transformed to topological overlap matrix (TOM) for estimating network connection, and hierarchical clustering was used to classify genes. Different colors in the cluster tree represented different gene modules. Genes were assigned into modules mainly according to weighted correlation of their expression patterns.

### 2.4 GO and KEGG functions analysis

Functional annotation of intersected targets was performed by the R package ClusterProfiler. Gene Ontology (GO) and Kyoto Encyclopedia of Genes and Genomes (KEGG) were used to understand the functional categories and pathway meaning of these genes (13, 14). GO terms and KEGG pathways with both p value and q value lower than 0.05 were treated as significant results.

### 2.5 Screening of hub genes by LASSO

LASSO is a shrinkage estimation method. It builds a penalty function that can make part of coefficients smaller and make some coefficients become zero. Therefore, it can combine feature selection and biased estimation, especially when data have complicated collinearity. In this study, Lasso logistic regression was used for selecting disease diagnostic marker features with the glmnet package (15).

### 2.6 Construction of nomogram model

The nomogram model was constructed on the basis of regression analysis. According to gene expression level, different variables were displayed on the same plane with scaled line segments, so the relationship among prediction factors could be shown visually (16). In a multivariable regression model, each factor level was given score according to the regression coefficient.

### 2.7 Analysis of immune cell infiltration

CIBERSORT is a commonly used method for estimating immune cell types in the microenvironment. It is based on support vector regression (17). In this study, CIBERSORT was applied to patient expression data, so as to infer the relative proportion of 22 immune infiltrating cells. Then, correlations between gene expression and immune cell content were calculated.

### 2.8 Gene set enrichment analysis

Gene set enrichment analysis (GSEA) was adopted to compare pathway differences (18). The annotated gene set version 7.0 from MSigDB was used as the background of subtype pathways. Enrichment of pathways was evaluated, and gene sets with adjusted P value < 0.05 were considered significant and then ordered according to consistency score.

### 2.9 Gene set variation analysis

Gene set variation analysis (GSVA) is a non-parametric and unsupervised approach for evaluating enrichment of transcriptomic gene sets (19). In the present study, gene sets were obtained from Molecular Signatures Database. The GSVA algorithm was used to calculate a comprehensive score for each gene set, which may reflect possible biological function changes among different samples.

### 2.10 MiRNA analysis

MiRNAs are small non-coding RNAs. They can regulate gene expression through promoting mRNA degradation or inhibiting mRNA translation (20). We searched miRNAs connected with key genes in the miRcode database (https://mirdb.org/), and then visualized the gene-miRNA network by Cytoscape software.

### 2.11 Transcriptional regulation analysis of hub genes

Transcription factors were predicted by using the R package RcisTarget. The calculation of RcisTarget depends on motifs. For each motif, the normalized enrichment score (NES) is influenced by the total number of motifs in database. To estimate motif over-representation in the gene set, the area under curve (AUC) was first calculated for each motif-motif set pair according to the recovery curve of motif ranking. And NES of every motif was calculated according to the AUC distribution in the whole gene set.

### 2.12 Single-cell data quality control, dimensionality reduction, clustering, and annotation

Expression profiles were imported into Seurat. Cells were filtered according to the total number of UMIs, the number of expressed genes, and the percentage of mitochondrial expression. Median absolute deviation (MAD) was used for quality control, and variables beyond 3 MAD from the median were regarded as outliers and removed. DoubletFinder (V2.0.4) was applied to remove doublet cells in each sample. LogNormalize was used to standardize total cell expression to 10,000 and then log transformation was performed. CellCycleScoring was used to calculate cell-cycle score. FindVariableFeatures was used to find highly variable genes. ScaleData removed variation caused by mitochondrial and ribosomal gene expression ratios and cell-cycle differences. RunPCA was used for linear dimensionality reduction and for selecting principal components. Harmony was further used to reduce batch effect, and RunUMAP was used for non-linear dimensionality reduction. CellMarker, PanglaoDB database, published literature, and SingleR software were together used to annotate cell types and marker genes.

### 2.13 Ligand-receptor interaction analysis

CellChat was used to infer and analyze cell-cell communication networks from single-cell data. It uses network analysis and pattern recognition to predict signal input, output, and functional coordination among cells (21). In this work, we examined interactions between cells and quantified the closeness of interaction by interaction weight and number, so as to evaluate cell activity or influence in disease state.

### 2.14 Pseudo-temporal analysis

Single-cell analysis can help to reveal transcriptional regulation during complicated physiological processes and in heterogeneous cell populations. It can also help to find genes in specific cell subtypes, intermediate biological states, and cell fate transition (22). Monocle places individual cells along pseudo-time trajectories by using asynchronous cellular processes. In this way, cell ordering may be related to biological processes such as differentiation.

### 2.15 Cmap drug prediction

Connectivity Map (CMap), developed by Broad Institute, contains gene expression profiles induced by different interventions. It can reveal functional links among small molecules, genes, and disease states (23). In this study, disease-related DEGs were used to predict candidate drugs against the disease condition.

### 2.16 Molecular docking

For the key genes, corresponding three-dimensional protein structures were obtained from AlphaFold database (https://alphafold.com/). Candidate drugs related to key genes were predicted from CMap and related resources, and chemical structures of compounds were accessed through PubChem (https://pubchem.ncbi.nlm.nih.gov/). AutoDock software was used for molecular docking, and genetic algorithm was selected with 50 docking runs. The docking pose with the lowest binding energy was selected for display. The final result was imported into PyMOL to show the binding sites between small molecules and proteins.

### 2.17 Molecular dynamics simulations

Molecular dynamics (MD) simulation was performed by Gromacs2023. GAFF force field was used for small molecules, AMBER14SB force field for proteins, and TIP3P water model for solvent. Protein and ligand files were merged to build complex simulation systems (24). Simulations were conducted under constant temperature and pressure as well as periodic boundary conditions. Hydrogen bonds were constrained by the LINCS algorithm, and integration step was 2 fs. Electrostatic interaction was calculated through Particle-mesh Ewald (PME) method with cut-off of 1.2 nm, while non-bonded interaction used 10 Å cut-off and was updated every 10 steps. Temperature was controlled at 298 K by V-rescale coupling, and pressure was kept at 1 bar by Berendsen method. NVT and NPT equilibration were each performed for 100 ps at 298 K. Then 100 ns MD simulation was conducted, and conformations were saved every 10 ps. After simulation, trajectories were analyzed by VMD and PyMOL. Stability was estimated by RMSD, Rg, RMSF, and buried SASA analyses.

### 2.18 RNA isolation and quantitative real-time PCR

Quantitative real-time PCR (qPCR) was used to measure mRNA expression of key genes in normal controls (n = 5), RA patients (n = 5), RA-TB patients (n = 5), and TB patients (n = 5). All samples were obtained from peripheral blood. Each sample was prepared in triplicate wells. GAPDH was used as internal reference gene, and the 2-ΔΔCt method was used to calculate mRNA fold change and normalize data across samples. Primers for MARCO were: forward primer, 5’-ATCAATGTTCCAAAGCCCAAGAGG-3’; reverse primer, 5’-GCGGTGAGCAGGATCAGGTAG-3’. Primers for ZBTB20 were: forward primer, 5’-AGAGAAGCAATGAAGTGGAGATGG-3’; reverse primer, 5’-GTTGACCGAAGGCTGTTGTAGG-3’.

### 2.19 Statistical analysis

All statistical analyses were completed by R language (version 4.2.2). All statistical tests were two-sided. *P* < 0.05 was considered to have statistical significance.

## 3 Results

### 3.1 Identification of differentially expressed genes in RA and TB

**Figure 1:**
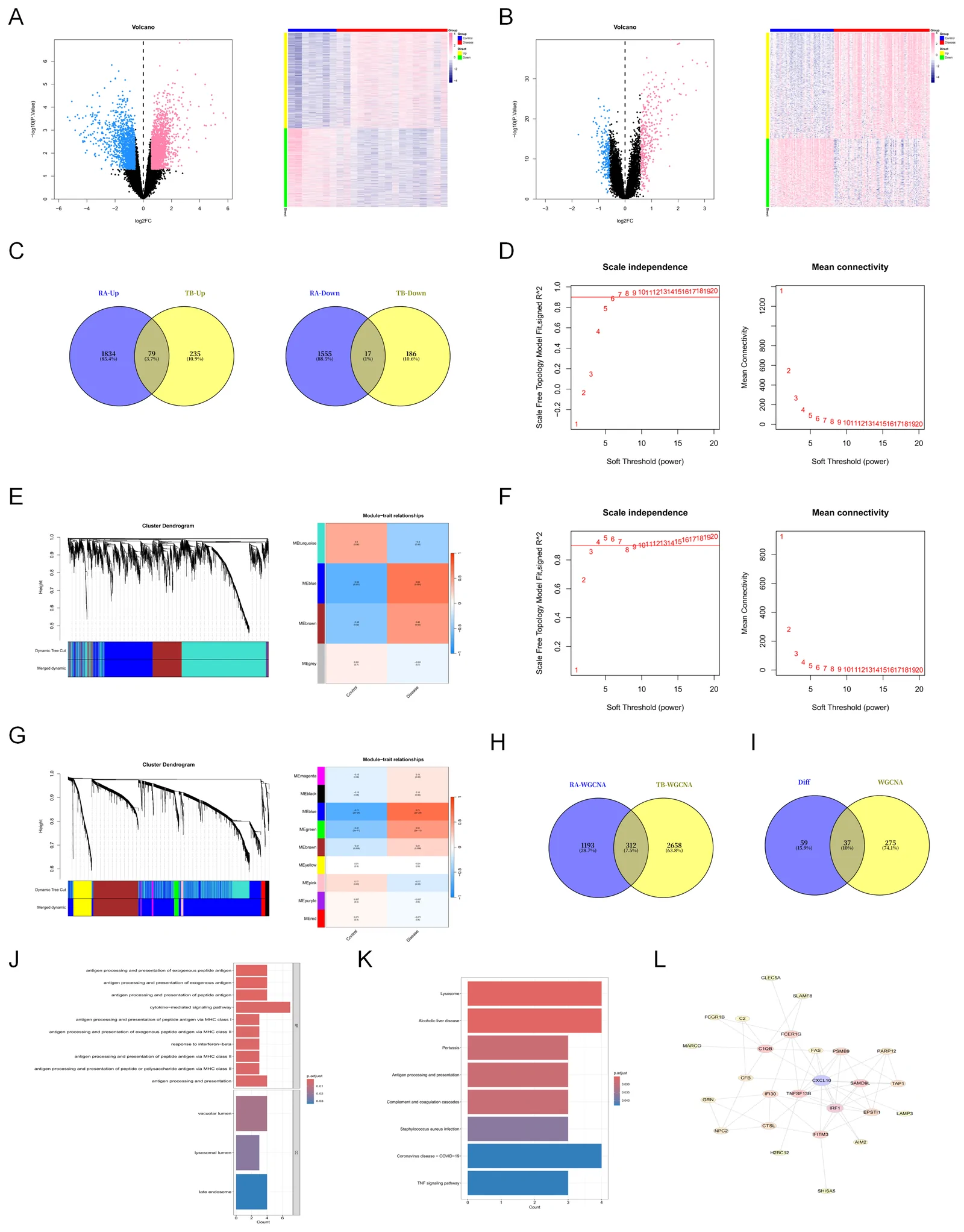
Identification of RA and TB differentially expressed genes, weighted gene co-expression network analysis, related functional enrichment analysis and PPI network construction. **(A)** Volcano and heat maps of GSE77298 differentially expressed genes. **(B)** Volcano and heat maps of GSE83456 differentially expressed genes.Red: up-regulated; blue: down-regulated. **(C)** Overlay of DEGs of GSE77298 and GSE83456. **(D)** Network topology analysis of RA. **(E)** Originating modules shown under the clustering tree and module-trait relationships of RA. **(F)** Network topology analysis of TB. **(G)** Originating modules shown under the clustering tree and module-trait relationships of TB. **(H)** 312 RA and TB gene overlaps identified by WGCNA. **(I)** 37 genes from the intersection of 312 genes with 96 differential genes. **(J)** GO analysis of these overlapping genes. **(K)** KEGG pathway enrichment analysis of these overlapping genes. **(L)** PPI network of characterized genes.

The RA-related data set GSE77298 was analyzed by Limma package. Totally, 3,485 differentially expressed genes were obtained. According to log-fold change (logFC), 1,913 genes were up-regulated and 1,572 genes were down-regulated, which were shown in volcano plot. Heatmap was also used to present their expression pattern (**Figure 1A**). For the TB data set GSE83456, 517 differentially expressed genes were identified, including 314 up-regulated and 203 down-regulated genes. Their expression pattern was shown by heatmap as well (**Figure 1B**). Between the two data sets, there were 79 commonly up-regulated genes and 17 commonly down-regulated genes showing expression difference (**Figure 1C**).

### 3.2 Weighted gene co-expression network analysis of RA and TB

To look for important genes in RA and TB cohorts, WGCNA networks were further built from expression profile data. In the GSE89632 data set, soft threshold β was selected as 6 (**Figure 1D**), and gene modules were then detected using the TOM matrix. Four gene modules were obtained (**Figure 1E**), and the blue module had the strongest relation with disease (cor = 0.64, p = 0.001). In GSE83456, the soft threshold β was 3 (**Figure 1F**), and 9 modules were identified by the same method (**Figure 1G**). The blue module was also most related with disease in this data set (cor = 0.71, p = 2e-24). After intersecting the genes from the most disease-related modules of two data sets, 312 common genes were collected (**Figure 1H**). These 312 genes were further overlapped with the 96 common differential genes, and 37 final intersection genes were obtained (**Figure 1I**).

### 3.3 Go and KEGG enrichment analysis and PPI network construction

Pathway analysis was then carried out for the 37 intersected genes. GO enrichment results indicated that these genes were mainly connected with cytokine-mediated signaling pathway, regulation of cell-cell adhesion, and cytoplasmic vesicle lumen (**Figure 1J**). KEGG analysis suggested enrichment in lysosome, antigen processing and presentation, and TNF signaling pathway (**Figure 1K**). Moreover, a protein-protein interaction network of these common targets was built through STRING database (http://cn.string-db.org) and visualized by Cytoscape (**Figure 1L**). These results imply that the interaction among these genes may participate in complicated molecular processes of RA and TB co-occurrence.

### 3.4 LASSO feature regression, external validation and ROC curve analysis

**Figure 2:**
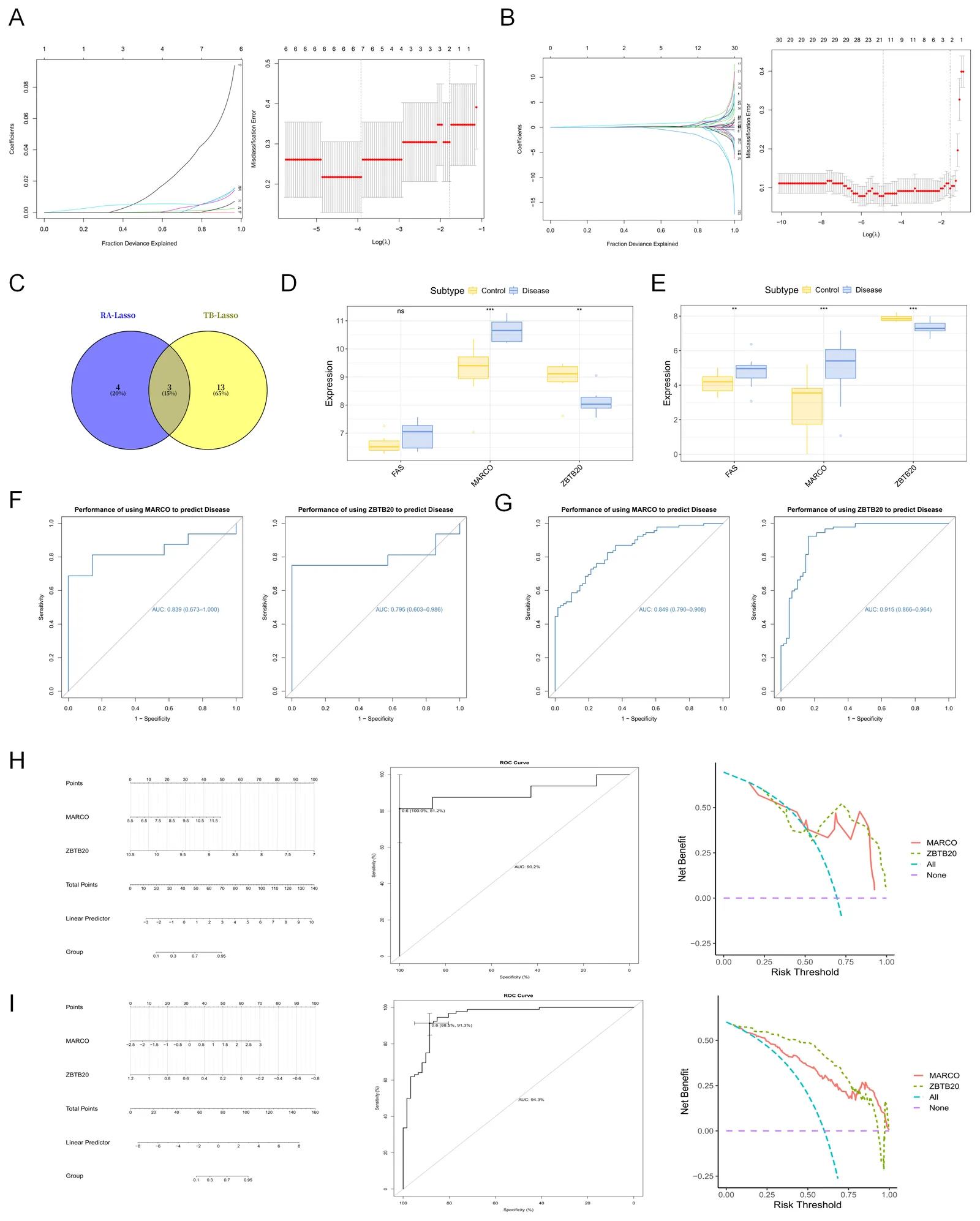
Identification of hub gene associated with RA and TB. **(A)** LASSO selects tuning parameters to verify performance of RA. **(B)** LASSO selects tuning parameters to verify performance of TB. **(C)** Cross-over genes for RA and TB were screened by LASSO. **(D)** External validation of FAS, MARCO and ZBTB20 in RA. **(E)** External validation of FAS, MARCO and ZBTB20 in TB. **(F)** ROC curves for MARCO (AUC = 0.839) and ZBTB20 (AUC = 0.795) in RA. **(G)** ROC curves for MARCO (AUC = 0.849) and ZBTB20 (AUC = 0.915) in TB. **(H)** Construction of Nomogram model, ROC and DCA curves of MARCO and ZBTB20 in RA. **(I)** Construction of Nomogram model, ROC and DCA curves of MARCO and ZBTB20 in TB.

To screen genes that may influence RA and TB at the same time, the intersected genes obtained above were used for LASSO regression (**Figures 2A, B**). LASSO analysis selected 7 characteristic genes in RA and 16 characteristic genes in TB. After overlapping the characteristic genes from the two diseases, 3 common genes were found, including *FAS*, *MARCO*, and *ZBTB20* (**Figure 2C**). These genes were further checked in external validation data sets. In GSE55235 for RA, *MARCO* and *ZBTB20* showed significant difference between groups (**Figure 2D**). In GSE19444 for TB, FAS, *MARCO*, and *ZBTB20* all showed significant differential expression (**Figure 2E**). Based on these results, *MARCO* and *ZBTB20* were used as key genes in following analysis. ROC curves were then applied to evaluate their diagnostic value. In RA, AUC values of *MARCO* and *ZBTB20* were 0.839 and 0.795 (**Figure 2F**). In TB, their AUC values were 0.849 and 0.915 (**Figure 2G**). These results show that *MARCO* and *ZBTB20* have relatively good predictive ability for disease occurrence and progression, and may be useful biomarkers.

### 3.5 Construction of nomogram model

Regression analysis results of hub genes were displayed by a nomogram model according to their expression levels. The regression result suggested that RA and TB samples contributed differently to the score distribution of hub gene expression. We then used ROC and DCA curves to verify the diagnostic effect of the two key genes in RA (**Figure 2H**) and TB (**Figure 2I**). The model indicated that both key genes had good capacity for predicting disease onset and development.

### 3.6 Immune infiltration analysis

**Figure 3:**
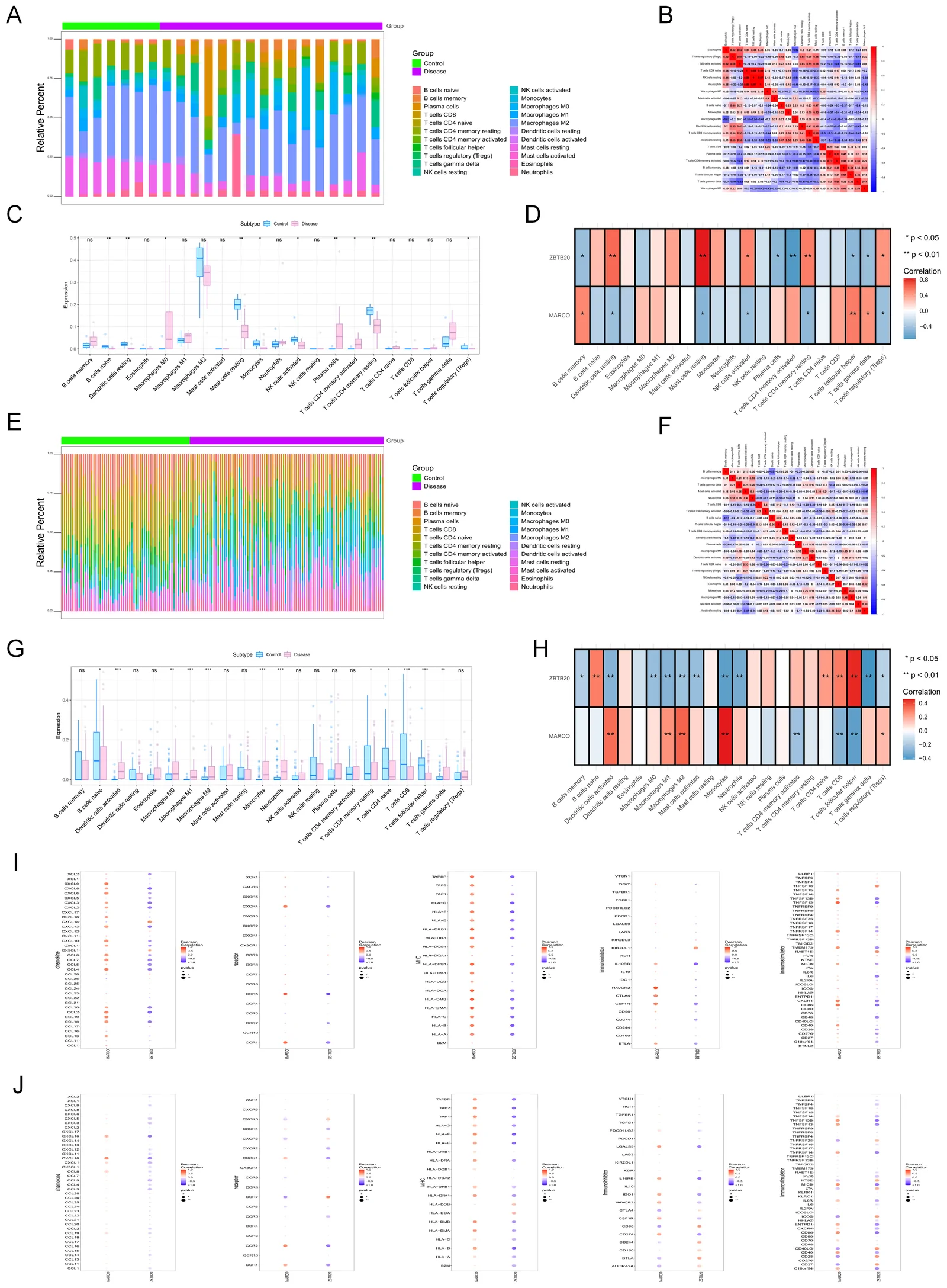
Correlation analysis between hub gene expression and immune infiltrations. **(A)** Immune cell infiltration level distribution in RA. **(B)** Correlation matrix between the proportion of immune cells in RA. Red color indicates positive correlation and blue color indicates negative correlation. **(C)** Composition of immune cell infiltrates in RA. **(D)** Composition of hub genes infiltrates in RA. **(E)** Immune cell infiltration level distribution in TB. **(F)** Correlation matrix between the proportion of immune cells in TB. **(G)** Composition of immune cell infiltrates in TB. **(H)** Composition of hub genes infiltrates in TB. **(I)** Correlation of key genes with chemokines,receptors,major histocompatibility complex(MHC), immunosuppressive and immunostimulatory factors in RA. **(J)** Correlation of key genes with chemokines,receptors,major histocompatibility complex(MHC), immunosuppressive and immunostimulatory factors in TB.*P < 0.05, **P < 0.01, ***P < 0.001.

Because the immune microenvironment is important for disease diagnosis, prognosis, and treatment response, immune infiltration was analyzed. In RA, the distribution and correlation of immune infiltration were shown in **Figures 3A, B**. Compared with controls, the disease group presented higher Macrophages M0, Plasma cells, and activated memory CD4 T cells, but lower naive B cells, resting dendritic cells, resting mast cells, monocytes, activated NK cells, resting memory CD4 T cells, and Tregs (**Figure 3C**). *MARCO* was positively related to memory B cells, follicular helper T cells, and gamma delta T cells, but negatively related to resting memory CD4 T cells, Tregs, activated NK cells, resting dendritic cells, and resting mast cells. *ZBTB20* showed a nearly opposite tendency: it was positively connected with resting memory CD4 T cells, Tregs, activated NK cells, resting dendritic cells, and resting mast cells, while negatively connected with memory B cells, plasma cells, activated memory CD4 T cells, follicular helper T cells, and gamma delta T cells (**Figure 3D**). In TB, similar analysis was conducted (**Figures 3E, F**). The disease group had higher Macrophages M0, Macrophages M2, neutrophils, and gamma delta T cells, but lower naive B cells, resting memory CD4 T cells, naive CD4 T cells, and CD8 T cells (**Figure 3G**). *MARCO* was positively correlated with Tregs, monocytes, M1 macrophages, M2 macrophages, and activated dendritic cells, but negatively correlated with CD8 T cells, activated memory CD4 T cells, and follicular helper T cells. *ZBTB20* was positively correlated with naive B cells, CD8 T cells, naive CD4 T cells, and follicular helper T cells, while negatively correlated with memory B cells, Tregs, gamma delta T cells, monocytes, M0/M1/M2 macrophages, activated dendritic cells, activated mast cells, and neutrophils (**Figure 3H**). Key genes were also connected with chemokines, receptors, major histocompatibility complex (MHC), immune suppressive factors, and immune stimulatory factors, implying their role in immune infiltration and microenvironment regulation (**Figures 3I, J**).

### 3.7 Signaling pathways involving hub genes

**Figure 4:**
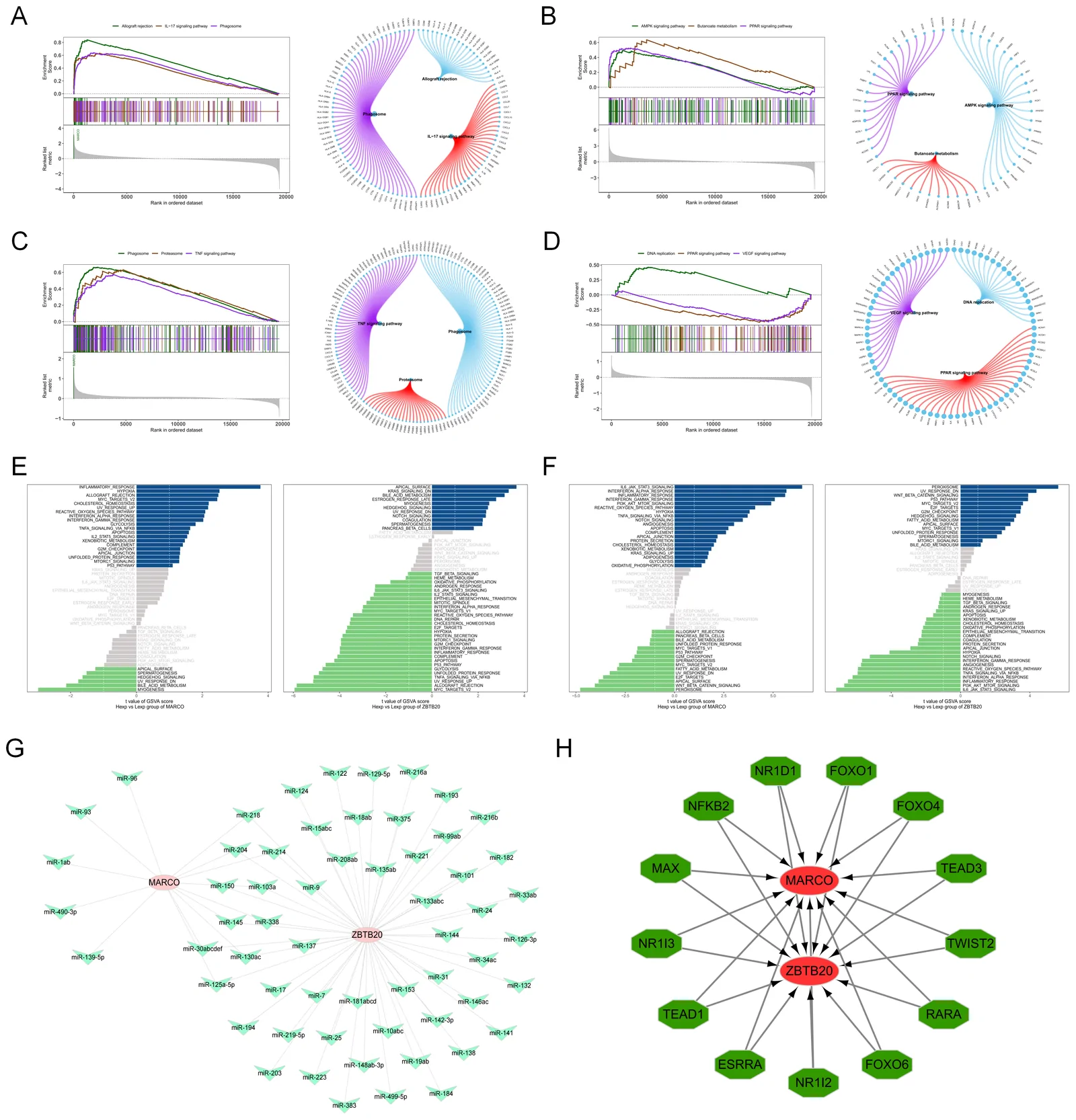
Signalling pathways, miRNA network construction and transcription factor regulatory networks involving key genes. **(A)** GSEA enrichment analysis of the gene MARCO in RA. **(B)** GSEA enrichment analysis of the gene ZBTB20 in RA. **(C)** GSEA enrichment analysis of the gene MARCO in TB. **(D)** GSEA enrichment analysis of the gene ZBTB20 in TB. **(E)** GSVA enrichment analysis of the gene MARCO and ZBTB20 in RA. **(F)** GSVA enrichment analysis of the gene MARCO and ZBTB20 in TB. **(G)** Construction of miRNA network associated with MARCO and ZBTB20 genes. **(H)** Transcription factor regulatory network of MARCO and ZBTB20 genes.

Specific signaling pathways related to hub genes were analyzed to explore how these genes may influence disease progression. In RA, GSEA showed that *MARCO* was enriched in allograft rejection, phagosome, and IL-17 signaling pathway (**Figure 4A**). *ZBTB20* was mainly connected with AMPK signaling pathway, PPAR signaling pathway, and butanoate metabolism (**Figure 4B**). In TB, *MARCO* was enriched in phagosome, TNF signaling pathway, and proteasome (**Figure 4C**), while *ZBTB20* was enriched in DNA replication, PPAR signaling pathway, and VEGF signaling pathway (**Figure 4D**). GSVA results in RA suggested *MARCO* enrichment in INFLAMMATORY_RESPONSE and HYPOXIA, and *ZBTB20* enrichment in APICAL_SURFACE and KRAS_SIGNALING_DN (**Figure 4E**). In TB, *MARCO* was enriched in IL6_JAK_STAT3_SIGNALING and INTERFERON_ALPHA_RESPONSE, and *ZBTB20* was enriched in PEROXISOME and UV_RESPONSE_DN (**Figure 4F**). These findings indicate that hub genes may affect disease process through these pathways.

### 3.8 MiRNA network construction and transcription factor regulatory network

Reverse prediction was conducted for the key genes through miRcode database. We obtained 59 miRNAs and 68 mRNA-miRNA interaction pairs, which were visualized by Cytoscape (**Figure 4G**). Taking the key genes as gene set, we also found that they could be regulated by several common transcription factors. Therefore, transcription factor enrichment was analyzed using cumulative recovery curve. Motif-TF annotation and important gene selection showed that the motif with highest normalized enrichment score was cisbp:M5613 (NES = 4.17). All enriched motifs and their corresponding transcription factors were presented in **Figure 4H**.

### 3.9 Single-cell sequencing of RA and TB

**Figure 5:**
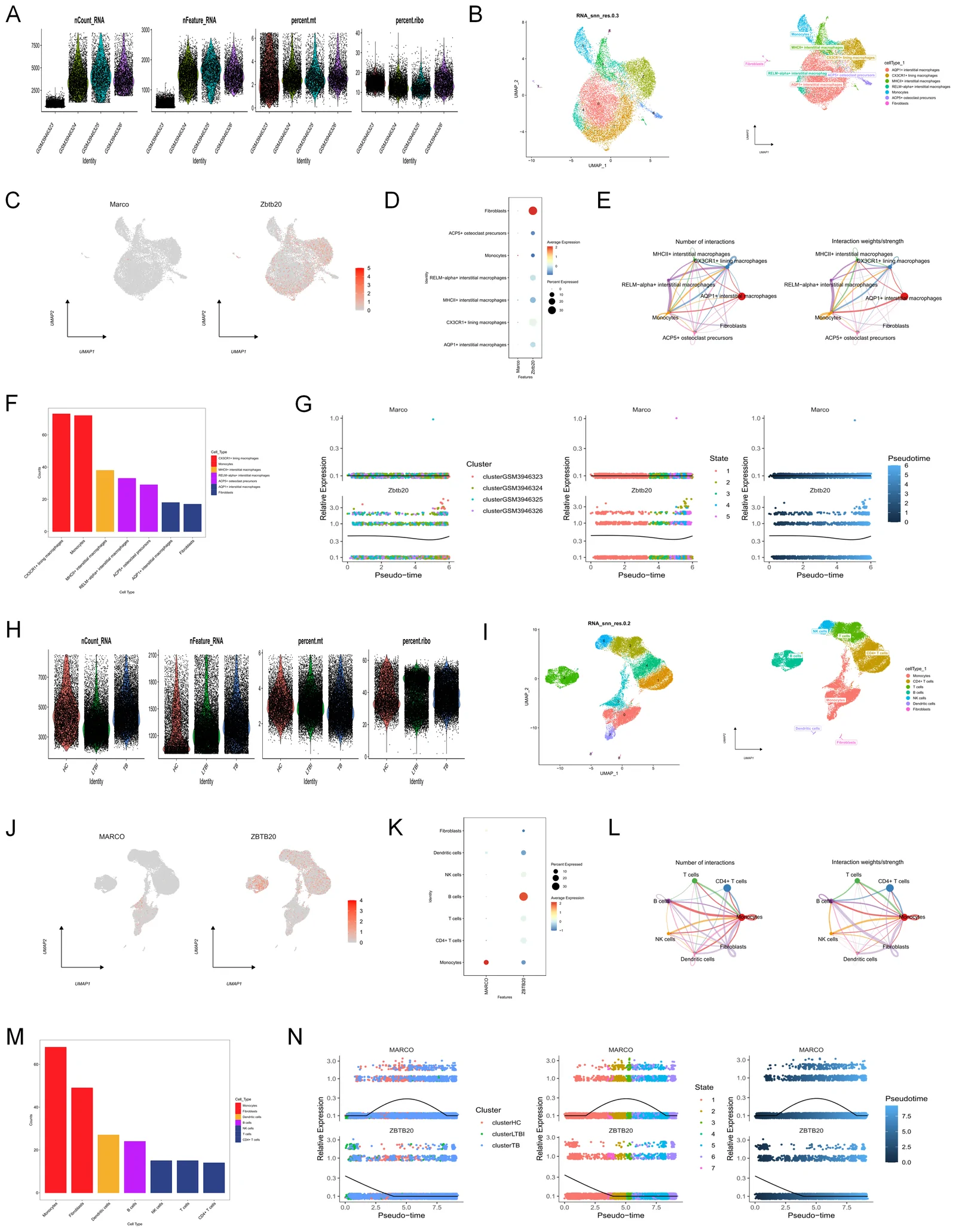
Single-cell dataset validates key gene expression of RA and TB. **(A)** Single-cell analysis of RA for initial screening and exclusion of low-quality data. **(B)** UMAP visualisation of cells in the RA single-cell RNA sequence dataset, with different colours indicating different clusters. **(C)** Distribution of key genes MARCO and ZBTB20 in RA single-cell RNA sequence dataset. **(D)** Expression of key genes MARCO and ZBTB20 in RA single-cell RNA sequence dataset. **(E)** Analysis of the number and interaction weight of ligand-receptor interactions in single-cell expression profiling analysis of RA. **(F)** Analysis of ligand-receptor interaction counts in single-cell expression profiling of RA. **(G)** Expression of key genes Marco and ZBTB20 in cell differentiation trajectories of RA. **(H)** Single-cell analysis of TB for initial screening and exclusion of low-quality data. **(I)** UMAP visualisation of cells in the TB single-cell RNA sequence dataset. **(J)** Distribution of key genes MARCO and ZBTB20 in TB single-cell RNA sequence dataset. **(K)** Expression of key genes MARCO and ZBTB20 in TB single-cell RNA sequence dataset. **(L)** Analysis of the number and interaction weight of ligand-receptor interactions in single-cell expression profiling analysis of TB. **(M)** Analysis of ligand-receptor interaction counts in single-cell expression profiling of TB. **(N)** Expression of key genes Marco and ZBTB20 in cell differentiation trajectories of TB.

For RA single-cell data quality control, cells with gene number lower than 200 were filtered out by criteria of nFeature_RNA > 200, percent.mt <= median + 3MAD, and nFeature_RNA/nCount_RNA <= median + 3MAD. After removing doublets by DoubletFinder, 15,955 cells were retained. Then 2,000 highly variable genes were selected, followed by normalization, homogenization, PCA, and Harmony analysis (**Figure 5A**; **Supplementary Figures 1A–C**). After UMAP dimensionality reduction, cells were divided into 9 subpopulations and annotated into 7 main cell types using specific marker genes, including AQP1+, CX3CR1+, MHCII+, RELM-alpha+ macrophages, monocytes, ACP5+ osteoclast precursor cells, and fibroblasts (**Figure 5B**; **Supplementary Figures 1D, E**). The spatial distribution and expression pattern of *MARCO* and *ZBTB20* in RA single-cell data were shown in **Figures 5C, D**. CellChat analysis further indicated a complicated interaction network among cell subtypes, and CX3CR1+ lining macrophages showed stronger interactions (**Figures 5E, F**). By calculating similarities among cells, pseudo-time differentiation trajectories were constructed and displayed according to pseudo-time, cell status, and sample source (**Supplementary Figures 1F, G**). Especially, *Zbtb20* expression showed a dynamic pattern that decreased first and then increased during differentiation (**Figure 5G**).

For TB data, 34,314 cells were kept after filtering and doublet removal by the same criteria (**Figure 5H**; **Supplementary Figures 1H–J**). The same process was used to select 2,000 highly variable genes and to perform UMAP dimensionality reduction. Ten subpopulations were obtained and annotated as monocytes, CD4+ T cells, and 7 other cell classes (**Figure 5I**; **Supplementary Figures 1K, L**). Gene expression validation (**Figures 5J, K**) and CellChat analysis showed that monocyte interactions were most active (**Figures 5L, M**; **Supplementary Figures 1M, N**). Pseudo-time trajectory and temporal differential gene clustering indicated that *MARCO* expression increased at first and then decreased, whereas *ZBTB20* stayed continuously down-regulated (**Figure 5N**).

### 3.10 Cmap drug prediction

**Figure 6:**
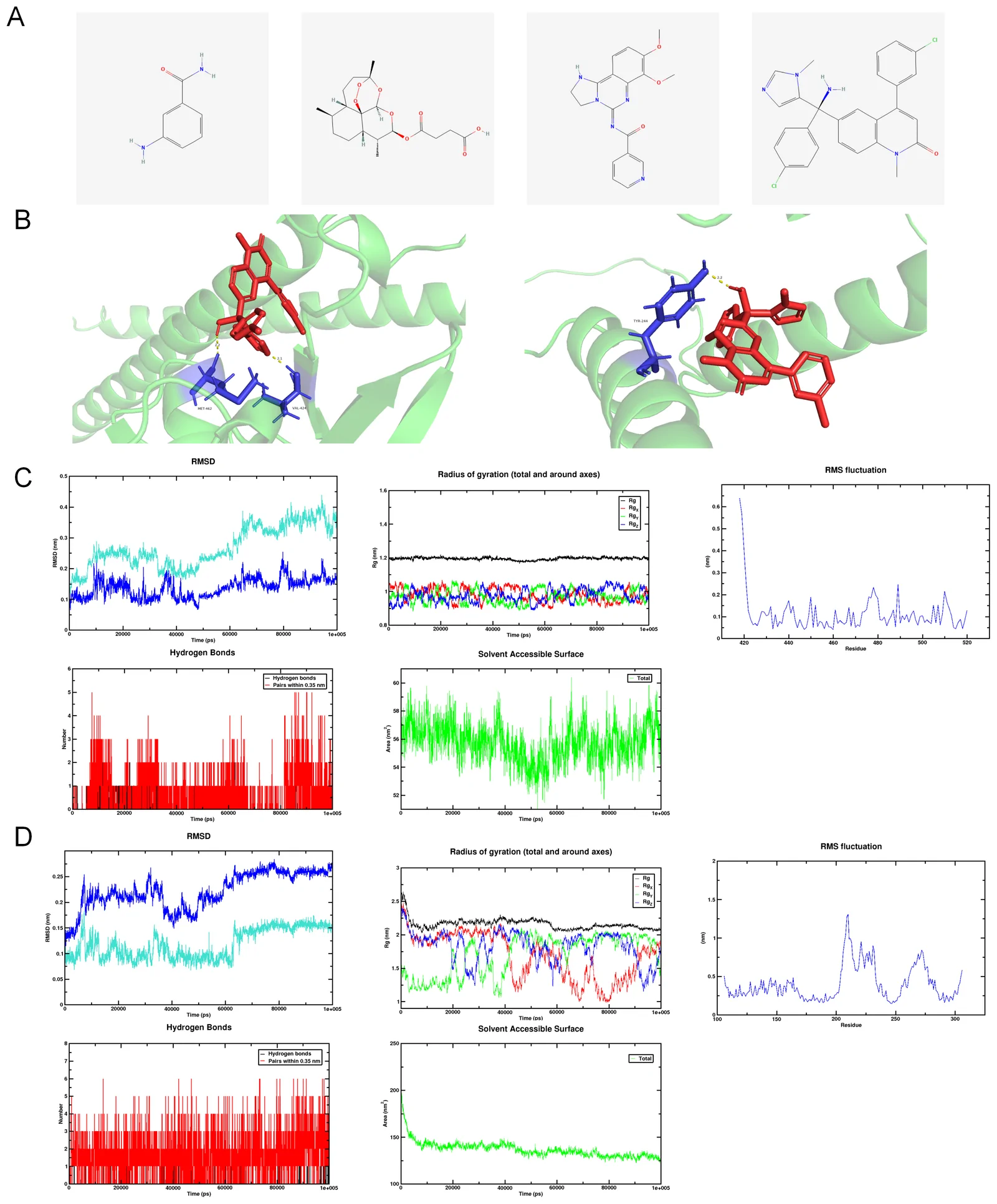
Drug prediction, molecular docking, molecular dynamics modelling in RA and TB. **(A)** Results of Drug prediction: 3-Aminobenzamide, Artesunate, PIK-90, Tipifarnib. **(B)** Molecular docking results of the key genes MARCO and ZBTB20. **(C)** Results of molecular dynamics simulations (MDS) involving Tipifarnib and MARCO. **(D)** Results of molecular dynamics simulations (MDS) involving Tipifarnib and ZBTB20.

To predict candidate drugs for RA and TB, commonly up-regulated and down-regulated intersected genes from the two diseases were divided into two groups and entered into Cmap database. The results showed that perturbation expression profiles of some drugs, including 3-Aminobenzamide, Artesunate, PIK-90, and Tipifarnib, were significantly and negatively correlated with the disease perturbation profile (**Figure 6A**). This result suggests that these compounds may serve as candidate therapeutic agents to relieve or even reverse the disease state.

### 3.11 Molecular docking of the key genes

The selected protein-compound pairs were *MARCO*: Q9UEW3-Tipifarnib and *ZBTB20*: Q9HC78-Tipifarnib. Docking results showed that the binding energy of *MARCO* Q9UEW3 with Tipifarnib was -6.57 kcal/mol, and the binding energy of *ZBTB20* Q9HC78 with Tipifarnib was -5.95 kcal/mol (**Figure 6B**).

### 3.12 MD simulation of the key genes

The binding between protein and ligand was relatively stable in both *MARCO*-Tipifarnib and *ZBTB20*-Tipifarnib systems. In *MARCO*-Tipifarnib, stable RMSD, Rg of complex and protein, and RMSF with limited residue fluctuation all indicated structural stability. Stable hydrogen bonds and buried SASA also supported stable protein-ligand binding (**Figure 6C**). In *ZBTB20*-Tipifarnib, the RMSD and Rg of complex became stable after about 65,000 ps, and the binding site gradually reached a stable status. RMSF suggested residues 200–250 as a relatively flexible region, while most other regions were stable. Hydrogen bond and buried SASA results also suggested a firm binding state (**Figure 6D**).These in silico results provide preliminary structural basis for subsequent pharmacological validation of the candidate targets.

### 3.13 Expression of the key genes in clinical samples

**Figure 7:**
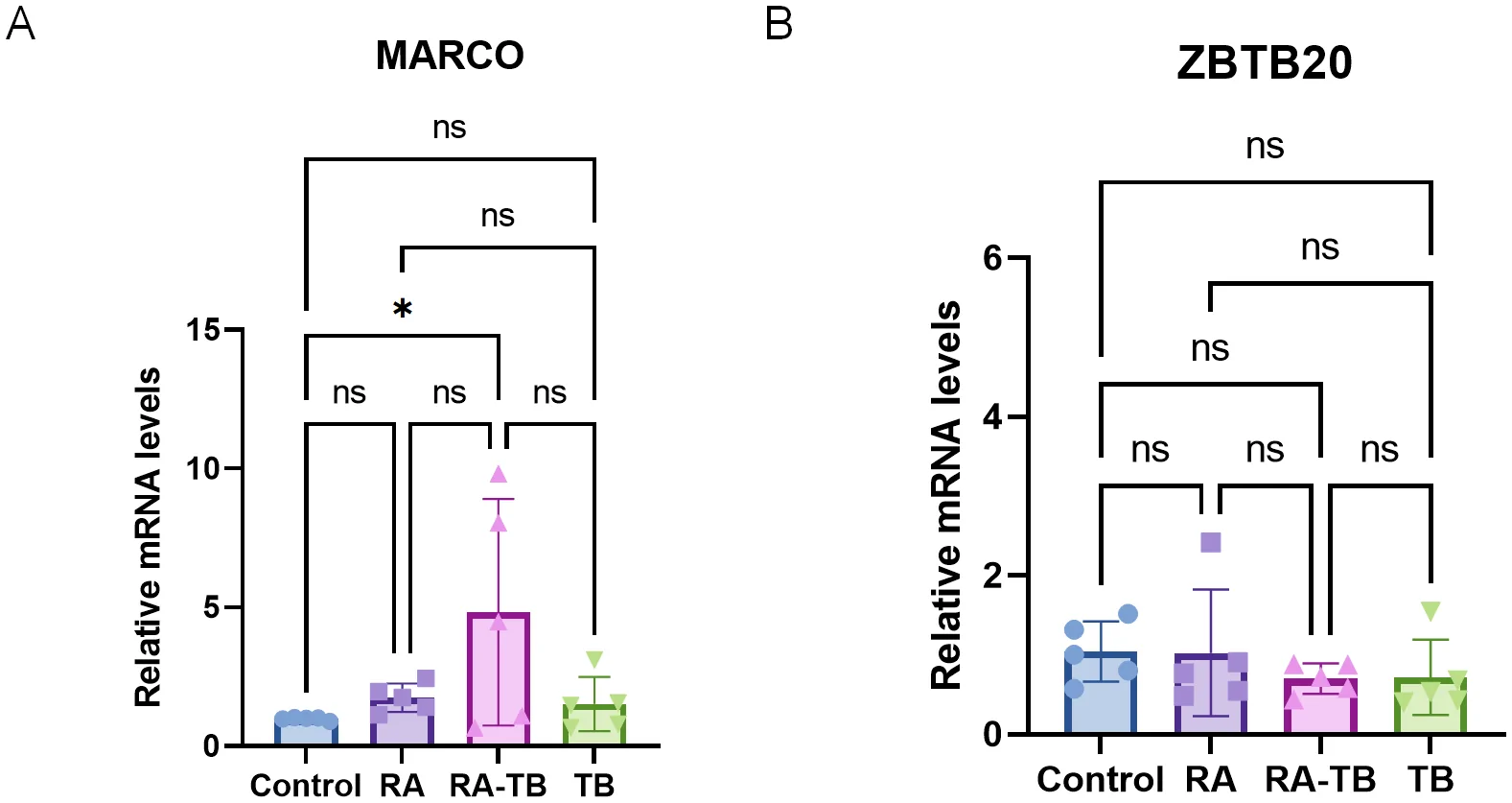
Clinical validation of key genes in RA and TB. **(A)** Expression levels of MARCO in Control, RA, RA-TB and TB blood samples. **(B)** Expression levels of ZBTB20 in Control, RA, RA-TB and TB blood samples.

Clinical sample data were statistically analyzed. In our small cohort, *MARCO* mRNA expression in the RA-TB group was significantly higher than that in the control group,while no statistically significant difference was observed in the RA or TB groups compared with controls. (P < 0.05, **Figure 7A**). However, *ZBTB20* expression did not show statistically significant difference among the four groups (**Figure 7B**).

## 4 Discussion

RA is a chronic autoimmune disease that mainly affects synovial joints and causes swelling, pain, and stiffness. TB is mainly caused by Mtb. More and more evidence has described a high occurrence of RA and TB comorbidity (25, 26). RA patients can benefit from immunosuppressive medicines, especially TNF pathway blockers, but this immune inhibition may also promote the change from LTBI to ATB (27, 28). Different clinical responses between RA and TB patients make precise treatment more difficult (29, 30). TB may negatively influence RA progression through many pathways, affecting disease activity, comorbidities, pain, quality of life, and mortality, and finally giving worse clinical outcomes. Also, RA and TB may interact with each other and form a vicious cycle that makes symptoms heavier. Previous studies were more focused on clinical and serological levels, and the genetic basis is still not very clear. Therefore, cellular and molecular studies are still needed, and patients with both diseases should be monitored carefully to reduce unnecessary harm.

In the present study, we used bioinformatics strategy aided by machine learning to investigate shared genetic and molecular mechanisms between RA and TB. Through common DEGs and co-expression module analysis, 312 candidate genes were first identified. Their relationship was then examined by WGCNA, PPI network analysis, and LASSO regression. *MARCO* and *ZBTB20* were selected as top hub genes, suggesting their possible roles in phagosome function and immune response. Although *ZBTB20* showed differential expression and diagnostic potential in public transcriptomic datasets, this finding was not replicated in our small clinical validation cohort (n = 5 per group). Similarly, although *MARCO* showed significantly higher expression in the RA-TB comorbidity group compared with healthy controls, it did not reach statistical significance in the single-disease groups (RA or TB) in our cohort. This inconsistency may arise from multiple factors, including small cohort size, disease heterogeneity, prior treatment exposure, and technical differences across detection platforms. More critically, we attribute the discrepancy primarily to the tissue-specific expression profiles of the target genes. This interpretation is further supported by the cellular localization and functional characteristics of the two genes. *MARCO*, a myeloid-restricted scavenger receptor, exhibits enriched expression in tissue-resident and lesion-associated macrophage populations, where it mediates pathogen sensing, phagocytic homeostasis, and inflammatory signal propagation (31). In inflamed synovial tissue, *ZBTB20* expression is tightly coupled to the activation state of immune and stromal cell compartments, including macrophages, lymphocytes, and fibroblast-lineage cells (32).Accordingly, the high AUC values observed in public datasets represent diagnostic performance specific to the tissue and pathological context from which they were derived, and highlight the potential of *ZBTB20* and *MARCO* as molecular indicators reflecting local tissue inflammation severity and lesion status. Moreover, in the absence of genetic perturbation experiments, their causal roles and therapeutic actionability remain unproven. Future validation studies should therefore evaluate their expression across diverse clinical specimen types and in larger, independent cohorts, with stratification according to disease activity, infection status, and treatment exposure, to more comprehensively determine their clinical utility.

*MARCO* is an important scavenger receptor encoded on chromosome 2. Its transmembrane protein can form trimers through collagenous domains and contains five domains: cytoplasmic, transmembrane, spacer, collagen, and SRCR (33). Direct evidence connecting *MARCO* with RA and TB is still limited. However, recent research has emphasized that *MARCO* participates in immune response regulation (34, 35) and inflammatory process (36, 37). When bacteria or viruses invade the body, *MARCO* may recognize and bind antigenic proteins or products expressed by pathogens. It can then activate signaling pathways in immune cells, such as macrophages and dendritic cells, and increased *MARCO* expression may promote inflammatory response. For example, loss of *MARCO* can inhibit ERK1/2 pathway and production of pro-inflammatory factors. *MARCO* also regulates cytokine secretion through TLRs, and inhibition of *MARCO* reduces TLR4/NF-kappaB activity and inflammation. In RA and TB, abnormal *MARCO* expression may impair bacterial clearance and worsen joint damage (38, 39). Our data suggest that *MARCO* may help to maintain inflammation in RA-TB comorbidity, in which abnormal expression may disturb bacterial elimination while aggravating joint injury.

The ZBTB protein family contains zinc finger and BTB domains and belongs to evolutionarily conserved transcription factors. These proteins usually have one N-terminal BTB domain and one or more C-terminal Cys2His2 (C2H2)/Krüppel-type zinc finger domains (40). At least 49 ZBTB proteins have been identified in human genome, and they have different functions in normal and disease conditions. *ZBTB20*, first found in dendritic cells and also named DPZF, HOF, or ZNF288 (41), mainly acts as a transcriptional repressor. It participates in cell differentiation, developmental regulation, innate immunity, and metabolic homeostasis (42–45). As an important transcriptional regulator, it is involved in lymphoid development and differentiation, and therefore may influence immune response and inflammation. For instance, *ZBTB20* is needed for full activation of Toll-like receptor (TLR) signaling, which is a key pathway in innate immune defense. Mechanistically, *ZBTB20* can enhance NF-kappaB signaling by repressing Nfkbia (IkappaBalpha), a classical negative regulator of the NF-kappaB pathway. This repression increases production of pro-inflammatory cytokines and type I interferons in TLR-stimulated macrophages (44). Taken together, *ZBTB20* may be a multi-function regulator of immune response and inflammation in the situation of RA-TB comorbidity.

The identification of *MARCO* and *ZBTB20* as candidate regulators of rheumatoid arthritis–tuberculosis (RA–TB) comorbidity may have potential clinical and translational relevance. Assessment of these genes, particularly when combined with established clinical, microbiological, imaging, or inflammatory markers, may contribute to the identification of patients at increased risk of RA–TB comorbidity. For example, integrating *MARCO* with established clinical or molecular biomarkers may improve the accuracy of RA risk stratification (46), whereas dysregulated *ZBTB20* expression may reflect an increased inflammatory burden and therefore provide additional information for disease diagnosis and progression assessment (47). Nevertheless, the diagnostic performance and clinical utility of these biomarkers require further validation in independent and well-characterized patient cohorts.

To identify potential pharmacological interventions for RA–TB comorbidity, we screened candidate compounds using the CMap database. Tipifarnib was identified as a potential candidate and was predicted, through molecular docking and molecular dynamics simulations, to form stable interactions with both *MARCO* and *ZBTB20*. Tipifarnib is a farnesyltransferase inhibitor whose principal mechanism of action involves disruption of protein prenylation and the subsequent modulation of downstream signaling pathways (48, 49). Previous studies have suggested that tipifarnib may influence prenylation-dependent pathways associated with NF-κB- and Toll-like receptor-mediated inflammatory responses, as well as cytokine-regulatory networks (50). These pathways are relevant to macrophage activation, immune regulation, and inflammatory responses implicated in both *MARCO* and *ZBTB20*, providing a plausible mechanistic basis for further investigation of tipifarnib in the context of RA–TB comorbidity. However, molecular docking and molecular dynamics simulations provide only computational predictions of potential ligand–target interactions and should not be interpreted as direct evidence of therapeutic efficacy. In particular, the present findings do not demonstrate that tipifarnib promotes mycobacterial clearance, suppresses the associated inflammation, or improves disease outcomes. Furthermore, without gene manipulation assays, the causal relationships between *MARCO/ZBTB20* and tipifarnib function, as well as the druggability of the two genes, remain unvalidated. Further functional assays including siRNA knockdown, CRISPR/Cas9 gene editing, and conditional knockout animal models are indispensable to clarify whether manipulating *MARCO* and *ZBTB20* reshapes infection status, immune profiles, and inflammatory phenotypes. Collectively, orthogonal biological models and experiments are warranted to validate Tipifarnib's actual bioactivity and translational therapeutic value.

Several limitations should be recognized. First, public data sets about RA and TB comorbidity were not enough, so sample size was restricted and causal relationship could not be fully analyzed. Larger samples and integrated comorbidity cohorts are necessary for stronger conclusion. Second, the clinical qRT-PCR cohort was small(n=5 per group), blood-based, and not paired with synovial or pulmonary/extrapulmonary lesion tissues, limiting direct comparison with public tissue datasets. Third, the detailed and complicated mechanisms by which *MARCO* and *ZBTB20* regulate RA and TB still need to be clarified. Fourth, all analyses presented herein are predominantly correlative and in silico-based. We have not conducted functional validations including siRNA-mediated gene knockdown, CRISPR gene editing, rescue assays, or knockout animal model experiments. Accordingly, causal relationships cannot be definitively established in this work, and the druggability of *MARCO* and *ZBTB20* as therapeutic targets remains unconfirmed. Lastly, results derived from CMap screening, molecular docking and molecular dynamics simulations are merely hypothesis-driven predictive data. Rigorous validation consisting of direct binding assays, target engagement detection, cellular functional tests and *in vivo* pharmacological studies is essential to verify these computational outcomes.

## 5 Conclusion

In conclusion, this work identified shared molecular signatures between RA and TB and suggested *MARCO* and *ZBTB20* as candidate diagnostic biomarkers. The findings provide a framework for further investigation of the shared molecular basis of RA and TB and suggest potential directions for biomarker development and drug discovery. However, validation in larger, tissue-resolved clinical cohorts and functional experimental models will be essential before these candidates can be translated into diagnostic or therapeutic applications.

## Data Availability

The original contributions presented in the study are included in the article/**Supplementary Material**. Further inquiries can be directed to the corresponding author.

## Supplementary material

Supplementary Figure 1 Single-cell sequencing of RA and TB. **(A)** Scatterplot of single cells from RA samples subjected to filtration. **(B)** 2000 highly variable genes in RA. **(C)** Standardised, PCA, harmonic analysis of RA. **(D)** Bubble diagrams of classical markers for 7 classes of cells of RA. **(E)** Histogram of the proportions of the 7 cell types in RA. **(F)** Constructing cell differentiation trajectories of RA.**G** Visualisation of the genomes with the largest changes along the proposed time difference in RA. **(H)** Scatterplot of single cells from TB samples subjected to filtration. **(I)** 2000 highly variable genes in TB. **(J)** Standardised, PCA, harmonic analysis of TB. **(K)** Bubble diagrams of classical markers for 7 classes of cells of TB. **(L)** Histogram of the proportions of the 7 cell types in TB. **(M)** Constructing cell differentiation trajectories of TB. **(N)** Visualisation of the genomes with the largest changes along the proposed time difference in TB.
